# Structure Elucidation and Toxicity Analyses of the Degradation Products of Aflatoxin B_1_ and Zearalenone by *Trichoderma reesei* GG-T40

**DOI:** 10.3390/jof12010046

**Published:** 2026-01-08

**Authors:** Yixuan Wang, Lixia Fan, Guidong Li, Changying Guo, Mingxiao Ning, Bingchun Zhang, Jiangyong Qu, Xianfeng Ren

**Affiliations:** 1 Institute of Agricultural Quality Standards and Testing Technology of Shandong Academy of Agricultural Sciences, Jinan 250100, China; 19811825257@163.com (Y.W.); superdemeter@163.com (L.F.); lguidong123@163.com (G.L.); cyguo808@163.com (C.G.); mxning428@163.com (M.N.); llzbest66@163.com (B.Z.); 2 College of Life Science, Yantai University, Yantai 264005, China; 3 Shandong Provincial Key Laboratory of Test Technology on Food Quality and Safety, Jinan 250100, China; 4 College of Food and Bioengineering, Henan University of Science and Technology, Luoyang 471003, China

**Keywords:** *Trichoderma reesei*, aflatoxin B_1_, zearalenone, biodegradation, detoxification, metabolic products, UPLC-Q-TOF MS

## Abstract

Mycotoxin contamination in agricultural products poses a serious challenge to food safety, severely threatening human and animal health and causing significant economic losses. This study aimed to investigate the degradation and detoxification capabilities of *Trichoderma reesei* GG-T40 against two representative mycotoxins—aflatoxin B_1_ (AFB_1_) and zearalenone (ZEN). The results showed that the degradation rates of AFB_1_ and ZEN by this strain reached 98.6% and 88.4%, respectively. Using ultra-performance liquid chromatography coupled with quadrupole time-of-flight mass spectrometry (UPLC-Q-TOF MS), the degradation products were systematically characterized, leading to the identification of six AFB_1_ degradation products (C_17_H_14_O_7_, AFD_1_: C_16_H_14_O_5_, C_11_H_10_O_4_, C_14_H_16_O_4_, C_15_H_10_O_4_, and C_17_H_14_O_5_) and two ZEN degradation products (α-ZOL and β-ZOL). Toxicity evaluation revealed that the key toxic structures of AFB_1_ were disrupted, significantly reducing or even eliminating the toxicity of its degradation products; ZEN was mainly converted into β-ZOL (accounting for 91.5%), which has lower estrogenic activity. Further toxicological experiments in mice confirmed that the degradation products were non-toxic and non-pathogenic under actual testing conditions, demonstrating systematic verification of their safety. In conclusion, *T. reesei* GG-T40 can efficiently and safely degrade AFB_1_ and ZEN, showing great potential for developing green control technologies for mycotoxin contamination in food and feed raw materials, with important application value for ensuring food safety.

## 1. Introduction

Mycotoxins are widely present in agricultural products such as grains and feed, posing a serious threat to global food security and often leading to significant food losses [1,2]. More critically, the co-contamination of multiple mycotoxins is becoming increasingly common, and their combined toxicity frequently exceeds the simple additive effects of individual toxins, substantially heightening health risks [3,4,5,6,7]. Among these, aflatoxin B_1_ (AFB_1_) and zearalenone (ZEN) have become focal points due to their high contamination rates and potent toxicity. AFB_1_ is not only a potent carcinogen but also causes damage to multiple organs including the liver, kidneys, nervous system, and reproductive system [8,9,10,11]; ZEN primarily exhibits strong reproductive toxicity and hepatorenal toxicity [12,13]. In China, the co-contamination of AFB_1_ and ZEN in feed is extremely severe, with exceptionally high contamination rates [14,15]. Exploring effective degradation methods to reduce their hazards is crucial for ensuring food safety and public health.

Existing physical or chemical detoxification methods are often accompanied by limitations such as poor specificity, unstable efficacy, nutrient loss, or secondary pollution [16]. In contrast, biodegradation methods offer advantages of high efficiency, specificity, and environmental friendliness [17]. Among these, fungi of the genus *Trichoderma*, particularly the industrially widely used *Trichoderma reesei*, are considered promising candidate strains due to their high safety profile and known effectiveness in degrading AFB_1_ [18,19,20]. Although there are currently no reports on the direct degradation of ZEN by *T. reesei*, given its efficient degradation capability for AFB_1_ and the documented effectiveness of other Aspergillus species in mycotoxin degradation [21,22,23], we speculated that *T. reesei* might also possess the potential to degrade ZEN. This speculation was supported in the preliminary screening of this study: we successfully screened one strain, *T. reesei* GG-T40, from 26 *Trichoderma* strains, which, compared to other *Trichoderma* strains, exhibited significant and stable degradation efficacy against both AFB_1_ and ZEN, indicating important development and application value.

**Figure 1:**
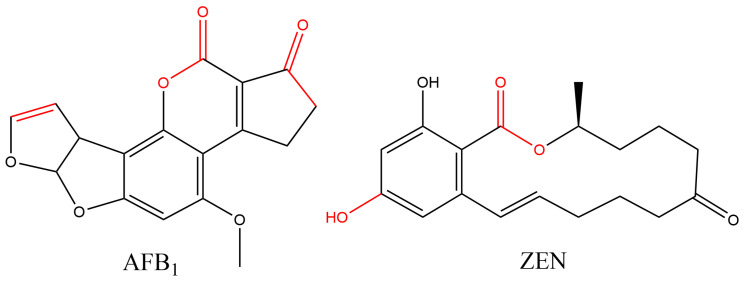
The Structures and Toxicity-Related Sites of AFB_1_ and ZEN.

Although many microorganisms, including *Trichoderma* species, have been confirmed to degrade both AFB_1_ and ZEN [3,24,25], their degradation mechanisms, particularly the specific structures of degradation products, metabolic pathways, and types of reactions involved, remain unclear. Due to the complexity of the matrix following microbial degradation, the isolation and identification of degradation products are extremely challenging, which significantly hinders progress in mechanistic studies. Nonetheless, elucidating the degradation mechanisms, characterizing the structures of degradation products, and assessing their safety are essential for promoting the application of microbial degradation technology in the food and feed industries. The key to achieving true detoxification lies in disrupting the critical toxic sites of the toxin molecules (Figure 1 illustrates the structures of AFB_1_ and ZEN and the distribution of their toxic sites).

Conventional analytical methods are often inadequate to address the challenges of identifying complex degradation products. Ultra-performance liquid chromatography coupled with quadrupole time-of-flight mass spectrometry (UPLC Q-TOF MS), with its high separation capability, high sensitivity, and powerful structural elucidation capability, provides an effective solution for this purpose [26,27,28,29]. Therefore, this study aims to utilize UPLC Q-TOF MS technology to systematically analyze the products generated during the degradation of AFB_1_ and ZEN by *T. reesei* GG-T40, to reveal their potential metabolic pathways and synergistic degradation mechanisms, and to preliminarily assess the alterations in toxicity of the degradation products. This research will provide an important theoretical foundation and scientific basis for developing safe and efficient mycotoxin biodegradation strategies based on *Trichoderma reesei*.

## 2. Materials and Methods

### 2.1. Chemicals and Strains

AFB_1_ and ZEN standards were purchased from Alta Scientific Co., Ltd. (Tianjin, China). Accurately weighed 1 mg of AFB_1_ or ZEN standard was dissolved in 10 mL of methanol (purity ≥ 99.95%) to obtain 100 μg/mL AFB_1_ and ZEN standard solutions, which were stored in brown glass vials at −18 °C. Acetonitrile and methanol (HPLC grade), ethyl acetate, and potato dextrose agar (PDA medium) were purchased from Macklin Biochemical Technology Co., Ltd. (Shanghai, China). Potato dextrose broth (PDB medium) was purchased from Haibo Biotechnology Co., Ltd. (Qingdao High-tech Industrial Park, Qingdao, China).

A total of 26 *Trichoderma* strains (Table 1), including *Trichoderma reesei* GG-T40, were provided by the Agricultural Product Quality and Safety Risk Assessment Laboratory, Shandong Academy of Agricultural Sciences (Jinan, Shandong Province, China), and the strains were stored in 25% glycerol/water at −80 °C.

### 2.2. Simultaneous Degradation of AFB_1_ and ZEN by Trichoderma Isolates

Mycelial plugs preserved in 25% glycerol/water solution were inoculated onto PDA plates and cultured at 28 ± 1 °C, 45% humidity, in darkness for 4–5 days. Subsequently, agar plugs of 6 mm diameter were cut from the colony edges using a cork borer and transferred to fresh PDA medium for activation.

AFB_1_ or ZEN standard solution was added to 10 mL of PDB medium for dilution to a final concentration of 200 ng/mL. Three mycelial plugs from pre-activated *Trichoderma* strains were obtained from colony margins using a cork borer and inoculated into the toxin-supplemented PDB medium. Meanwhile, PDB medium containing equivalent amounts of AFB_1_ or ZEN without fungal inoculation served as the control. The cultures were incubated at 29 °C with shaking at 175 rpm (SLY-1102C, Jintan Jingda Instrument Manufacturing Co., Ltd., Changzhou, China) for seven days. After the incubation period, 1 mL of culture medium was collected from each flask, filtered sequentially through filter paper and a 0.22-μm membrane, and analyzed by ultra-performance liquid chromatography–tandem mass spectrometry (UPLC–MS/MS) to determine the residual concentrations of AFB_1_ and ZEN.

### 2.3. The Degradation Products of AFB_1_ and ZEN by Trichoderma GG-T40

#### 2.3.1. Co-Culture of Trichoderma GG-T40 with AFB_1_ and ZEN

The procedure is similar to that described in Section 2.2 above, with the difference being that AFB1 or ZEN standard solution was added to 1000 mL of PDB medium and diluted to a final concentration of 200 ng/mL. Fifty pre-activated GG-T40 *Trichoderma* mycelial plugs were inoculated into toxin-supplemented PDB medium containing AFB_1_ or ZEN, respectively, with PDB medium containing an equivalent amount of toxin but no fungal inoculation serving as the control. The cultures were incubated at 29 °C with shaking at 175 rpm (SLY-1102C) for seven days, followed by filtration using medium-speed qualitative filter paper.

#### 2.3.2. Pre-Treatment Methods

The filtered solution was extracted with an equal volume of ethyl acetate three times to transfer the degradation products from the aqueous phase to the organic phase. The organic phase was further concentrated using a rotary evaporator (Heidolph VV2000, Heidolph, Shanghai, China) in a water bath at 60 ± 2 °C, and the solvent was removed under vacuum evaporation. The concentrate was redissolved with pure methanol.

The redissolved sample solution was placed in a 15 mL graduated centrifuge tube (KIMBLE^®^ 45500-15, Kimball Electronics, Nanjing, China) and concentrated to 2.0 mL using a nitrogen evaporator (Organomation^®^ N-EVAPTM 112, Organomation, Berlin, MA, USA) at a nitrogen flow rate of 0.3 L/min (99.999%, Praxair, Shanghai, China) in a water bath (Julabo^®^ F12, JULABO, Beijing, China) maintained at 40 ± 0.5 °C. The concentrate was precisely adjusted to a volume of 10 mL with methanol pre-chilled at 4 °C, and then the sample was filtered through a 0.22 μm membrane filter (Millipore^®^ SLGV033RB, Millipore, Darmstadt, Germany), and transferred into brown chromatography vials for UPLC-Q-TOF MS analysis.

#### 2.3.3. Operating Conditions for UPLC-Q-TOF MS

UPLC analysis was performed on a Waters ACQUITY UPLC-PDA system (Waters, Milford, MA, USA) equipped with an analytical reversed-phase C18 column (2.1 mm × 100 mm, 1.7 μm, ACQUITY BEH). The injection volume was 5 μL. The mobile phase consisted of 0.1% formic acid in water (phase A) and 0.1% formic acid in acetonitrile (phase B), with gradient elution conditions detailed in Table 2. The effluent from the UPLC system was introduced into the ESI-Q-TOF-MS detector at a flow rate of 0.4 mL/min.

Time-of-flight mass spectrometry detection was performed using a Waters Synapt Q-TOF system equipped with an electrospray ionization (ESI) source. AFB_1_ was analyzed in positive ion (PI) mode, while ZEN was analyzed in negative ion (NI) mode. The optimized conditions were set as follows: capillary voltage 3.0 kV, cone voltage 20 V, desolvation temperature 450 °C, cone gas flow rate 50 L/h, and desolvation gas flow rate 900 L/h. To obtain more information about parent and fragment ions, collision-induced dissociation was applied for each compound using collision energies (CE) of 10 eV or 40 eV. Full-scan MS analysis was conducted within the *m*/*z* range of 50–1200.

#### 2.3.4. Data Processing and Structure Analysis

The degradation rates of AFB_1_ and ZEN were calculated using the following formula (F1):

F1 (%) = (C1 − S1)/C1 × 100%

where S1 represents the toxin concentration in the experimental group inoculated with *Trichoderma* and supplemented with AFB_1_ or ZEN, and C1 represents the concentration of AFB_1_ or ZEN in the control group.

MassLynx V4.1 and MassFragment software V4.1 were employed for data processing and structural characterization of the products generated from the degradation of AFB_1_ and ZEN by *T. reesei* GG-T40, respectively. MassLynx can predict molecular formulas of potential components based on mass spectrometry data; however, this alone is insufficient to unambiguously identify the degradation products. MassFragment, as an intelligent chemical software for structural analysis, determines the structures of the degradation products by interpreting the precursor and fragment ion information derived from the data.

### 2.4. Analysis of α-/β-ZOL by HPLC

Separately prepare standard solutions of α-ZOL and β-ZOL at a concentration of 1 μg/mL each. Prepare a mixed standard solution containing 0.1 μg/mL α-ZOL + 0.1 μg/mL β-ZOL + 0.01 μg/mL ZEN for subsequent HPLC detection.

The analysis was performed using an LC-30A UPLC system (Shimadzu Corporation, Kyoto, Japan) coupled with a 4500 triple-quadrupole tandem mass spectrometer (AB Sciex; Framingham, MA, USA). The system was equipped with an analytical reversed-phase C-18 column, Poroshell 120 EC-C18 (100 mm × 4.6 mm, 2.7 μm) (Agilent Technologies Industries Co., Ltd., Santa Clara, CA, USA). The injection volume was 5 μL. The mobile phase consisted of 0.1% FA Water (mobile phase A) + 0.1% FA MeOH (mobile phase B). The gradient elution conditions are shown in Table 3.

### 2.5. Toxicity Analysis of Degradation Products

To systematically evaluate the in vivo toxicity of the degradation products, this study conducted acute oral toxicity tests and intraperitoneal injection pathogenicity tests in mice. The test sample consisted of culture supernatant containing AFB_1_/ZEN degradation products. AFB_1_ and ZEN were added to the fresh liquid medium of GG-T40 at a final concentration of 200 ng/mL each, and incubated under conditions of pH 5.5, 28 °C, and 175 rpm for 7 days. After cultivation, the culture broth was filtered to remove bacterial cells, and the supernatant was collected. The supernatant was then filtered through a 0.22 μm sterile membrane. To meet the high-dose requirement for the acute oral toxicity test, the sterile-filtered culture supernatant was subjected to lyophilization to obtain a lyophilized powder. Subsequently, the lyophilized powder was reconstituted with sterile physiological saline to prepare a homogeneous suspension for mouse oral gavage. For the intraperitoneal injection pathogenicity test, the aforementioned filtered liquid culture supernatant was used directly. For the acute oral toxicity test: ICR mice (20 mice, half male and half female) were used as one group. Prior to the experiment, the mice were fasted for 6 h with free access to water, followed by a single oral gavage of the sample at a dose of 0.4 mL/20 g body weight. After gavage, fasting continued for 2 h before normal feeding was resumed. The mice were observed continuously for 14 days, with daily records of survival status and toxic reactions. For the intraperitoneal injection pathogenicity test: one dose group was established, using ICR mice (40 mice, half male and half female). The mice were intraperitoneally injected with the sample at a dose of 0.2 mL/20 g body weight and observed daily. To assess pathological changes, 5 male and 5 female mice were euthanized on day 3 and day 7, respectively, for gross pathological examination; all remaining mice were euthanized at the end of the experiment on day 14 and subjected to dissection for gross pathological observation.

## 3. Results and Discussion

### 3.1. Degradation Rates of AFB_1_ and ZEN by Trichoderma Isolates

**Figure 2:**
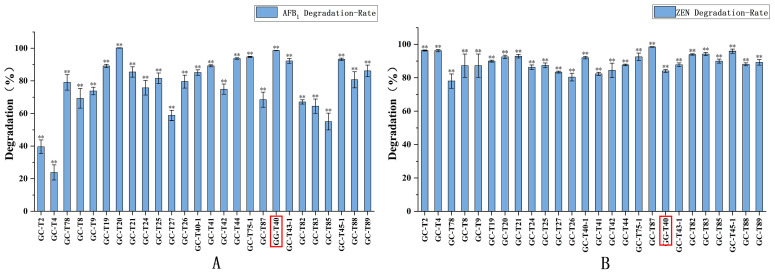
Degradation of AFB_1_ (**A**) and ZEN (**B**) by 26 *Trichoderma* strains. The representative strain *T. reesei* GG-T40 showed degradation rates of 98.6% for AFB_1_ and 88.4% for ZEN. Bars marked with “**” indicate a significant difference at the level of *p* < 0.01 (Tukey–Kramer multiple comparison test). Values represent the means of three replicates and their standard errors.

Previous studies have confirmed that a variety of microorganisms and their enzyme preparations possess significant degradation capabilities for AFB_1_ or ZEN. For instance, *Stenotrophomonas acidoaminiphila* [30], *Bacillus velezensis* [31], *Bacillus shackletonii* [32], *Lactobacillus plantarum* [33], *Saccharomyces cerevisiae* [34], as well as *Bacillus subtilis* and *Bacillus natto* [35] can effectively degrade AFB_1_ or ZEN, achieving degradation rates for single toxins ranging from 87% to 100% under suitable conditions. Further research indicates that key enzyme preparations obtained through isolation and purification often exhibit higher degradation efficiency and more stable catalytic performance. For example, Branà et al. [36] extracted a crude enzyme with laccase activity from the spent mushroom substrate of the edible fungus *Pleurotus eryngii*, which degraded 90% of AFB_1_ after condition optimization; Cheng et al. [37] utilized extracellular enzymes secreted by *Bacillus megaterium* HNGD-A6 as the active degradation substance, demonstrating a 94.66% AFB_1_ removal capacity; Shcherbakova et al. [38] heterologously expressed the ZHD101 gene in *Penicillium canescens* PCA-10, resulting in a secreted protease (PR-ZHD). This engineered enzyme maintained significant catalytic efficiency even under non-optimal temperatures, degrading 80–86% of ZEN within 5 h at 10 °C. However, microbial resources capable of simultaneously and efficiently degrading these two structurally distinct toxins, AFB_1_ and ZEN, remain relatively scarce [39,40,41]. In this study, preliminary screening of 26 *Trichoderma* strains revealed that fungi within this genus exhibit unique potential in this regard. As shown in Figure 2, multiple *Trichoderma* strains, including *T. citrinoviride* GC-T20 and *T. reesei* GG-T40, demonstrated efficient and simultaneous degradation of both AFB_1_ and ZEN. Among them, *T. reesei* GG-T40 degraded 98.6% of AFB_1_ and 88.4% of ZEN within 7 days. Notably, as a model strain for industrial enzyme production that has been extensively studied, *T. reesei* possesses outstanding characteristics such as rapid growth, mature fermentation processes, and a clear genetic background. Its degradation activity is expected to remain stable across a wider range of pH and temperature conditions, which is crucial for technological reproducibility and scale-up production. Furthermore, this strain has been recognized as a safe microorganism by regulatory agencies such as the U.S. FDA and does not produce known mycotoxins or antibiotics under production conditions, providing an important safety foundation for its future application in the food and feed sectors.

However, compared to the screening of degrading strains, research on the structure of degradation products, degradation pathways, and mechanisms remains relatively limited, leading to difficulties in practical application and potential safety concerns. Therefore, based on the preliminary screening and acquisition of the simultaneously efficient degrading strain *T. reesei* GG-T40, this study will further systematically investigate the molecular mechanisms underlying its degradation of AFB_1_ and ZEN, and conduct a comprehensive evaluation of the toxicological activity of the degradation products. This aims to provide a solid scientific basis for the safety and application feasibility of this strain.

### 3.2. Molecular Formulas of Degradation Products of AFB_1_ and ZEN

**Figure 3:**
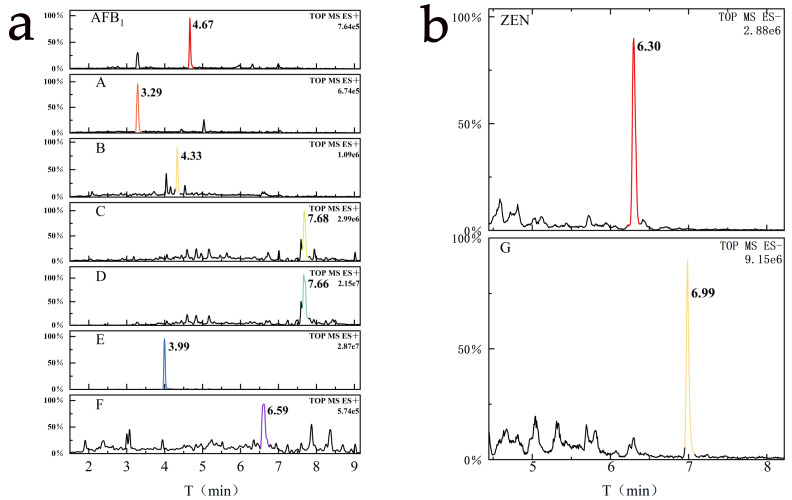
Chromatographic analysis of the degradation of AFB_1_ (**a**) and ZEN (**b**) by *T. reesei* GG-T40, and the corresponding chromatographic peaks of potential degradation products along with their relative abundance values.

Through systematic optimization of chromatographic conditions, including key parameters such as mobile phase composition, flow rate, column temperature, and detection wavelength, we successfully achieved effective separation of the components in the samples. As shown in Figure 3a, in the experimental group of *T. reesei* GG-T40 co-cultured with AFB_1_, six well-resolved and symmetrical chromatographic peaks were observed at retention times of 3.288, 4.330, 7.679, 7.665, 3.994, and 6.585 min. These six components (designated as products A–F) were preliminarily identified as potential degradation products of AFB_1_, based on high-resolution mass spectrometric analysis and comparison with reference standards. Similarly, as shown in Figure 3b, in the *Trichoderma*–ZEN co-culture group, a newly emerged chromatographic peak at 6.987 min was inferred to be a degradation product of ZEN (designated as product G).

Utilizing the MassLynx V4.1 software platform, in conjunction with Q-TOF high-resolution mass spectrometry data and based on previously accumulated degradation experience and key parameters such as Double Bond Equivalence (DBE), we conducted systematic screening and elemental composition inference of the degradation products of AFB_1_ and ZEN, preliminarily identifying the probable molecular formulas of each component. Table 4 and Table 5 detail the retention times, proposed elemental compositions, theoretical mass-to-charge ratios, measured mass-to-charge ratios, mass errors, and double bond equivalence values corresponding to each chromatographic peak of AFB_1_, ZEN, and their potential degradation products, respectively.

Among these, AFB_1_ (C_17_H_12_O_6_) showed a protonated ion [M + H]^+^ with *m*/*z* 313.0712 upon mass spectrometric analysis. Several characteristic ions detected exclusively in the *T. reesei* GG-T40 treatment group included: *m*/*z* 331.0818 (product A, C_17_H_15_O_7_), 287.0919 (product B, C_16_H_15_O_5_), 207.0657 (product C, C_11_H_11_O_4_), 249.1127 (product D, C_14_H_17_O_4_), 255.0657 (product E, C_15_H_11_O_4_), and 299.0919 (product F, C_17_H_15_O_5_), all detected in the form of [M + H]^+^. Similarly, for ZEN (C_18_H_21_O_5_), its deprotonated ion [M − H]^−^ had *m*/*z* 317.1389; while its degradation product G (C_18_H_23_O_5_) exhibited a corresponding ion [M − H]^−^ with *m*/*z* 319.1545. The above results indicate that *T. reesei* GG-T40 possesses certain metabolic transformation capabilities toward both AFB_1_ and ZEN. The structures of the related products require further investigation.

### 3.3. Structural Formulas of AFB_1_ Degradation Products

In traditional research, structural identification of unknown degradation products typically requires initial separation and purification of the target compounds using chromatographic and other methods to obtain high-purity samples. This is followed by the combined application of various spectroscopic techniques such as mass spectrometry (MS), ultraviolet spectroscopy (UV), infrared spectroscopy (IR), and nuclear magnetic resonance (NMR) [42,43], along with comparison with reference standards, to elucidate their chemical structures. However, in the present study, the metabolic products resulting from the degradation of AFB_1_ and ZEN by *T. reesei* GG-T40 are numerous, exhibit high structural complexity, and show significant background interference in mass spectrometry, making it difficult to obtain sufficiently pure samples for structural analysis via conventional micro-preparation methods. Therefore, this study adopted a targeted structure inference strategy based on high-resolution mass spectrometry: using MassLynx V4.1 software to collect accurate mass numbers of precursor and fragment ions provided by Q-TOF MS, combined with key parameters such as molecular formulas and Double Bond Equivalence (DBE) listed in Table 4 and Table 5, to systematically screen and infer possible structures of the degradation products. DBE is an important indicator characterizing the degree of unsaturation in a molecule (including the number of double bonds and ring structures). The DBE value of AFB_1_ itself is 11.5, while the DBE values of its six degradation products range between 6.5 and 10.5 (Table 4), all lower than that of AFB_1_, indicating the occurrence of double bond cleavage or ring-opening reactions during the degradation process, leading to a decrease in unsaturation. Based on literature reports, the degradation mechanisms of AFB_1_ often involve reaction types such as oxidation [44], hydrolysis, hydroxylation, and reduction [45]. On this basis, we preliminarily hypothesized possible structures of the degradation products and imported them in MOL format into MassFragment software to construct a degradation product structure library. This tool can prioritize candidate structures that match the mass spectrometry fragmentation patterns based on accurate mass matching rules, thereby enhancing the rationality and reliability of structural inference of the products.

**Figure 4:**
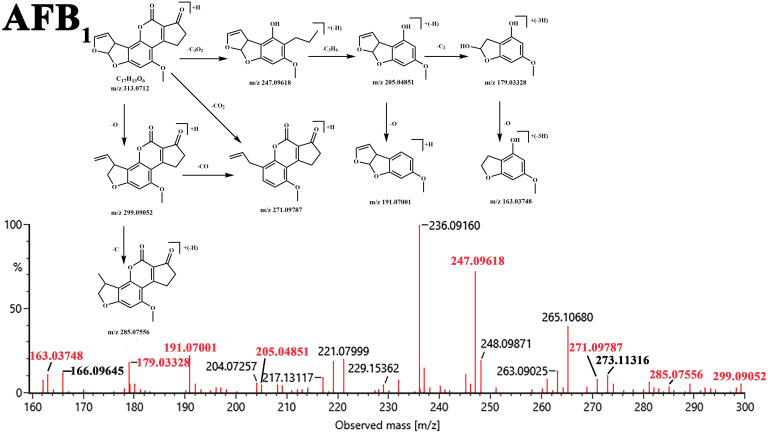

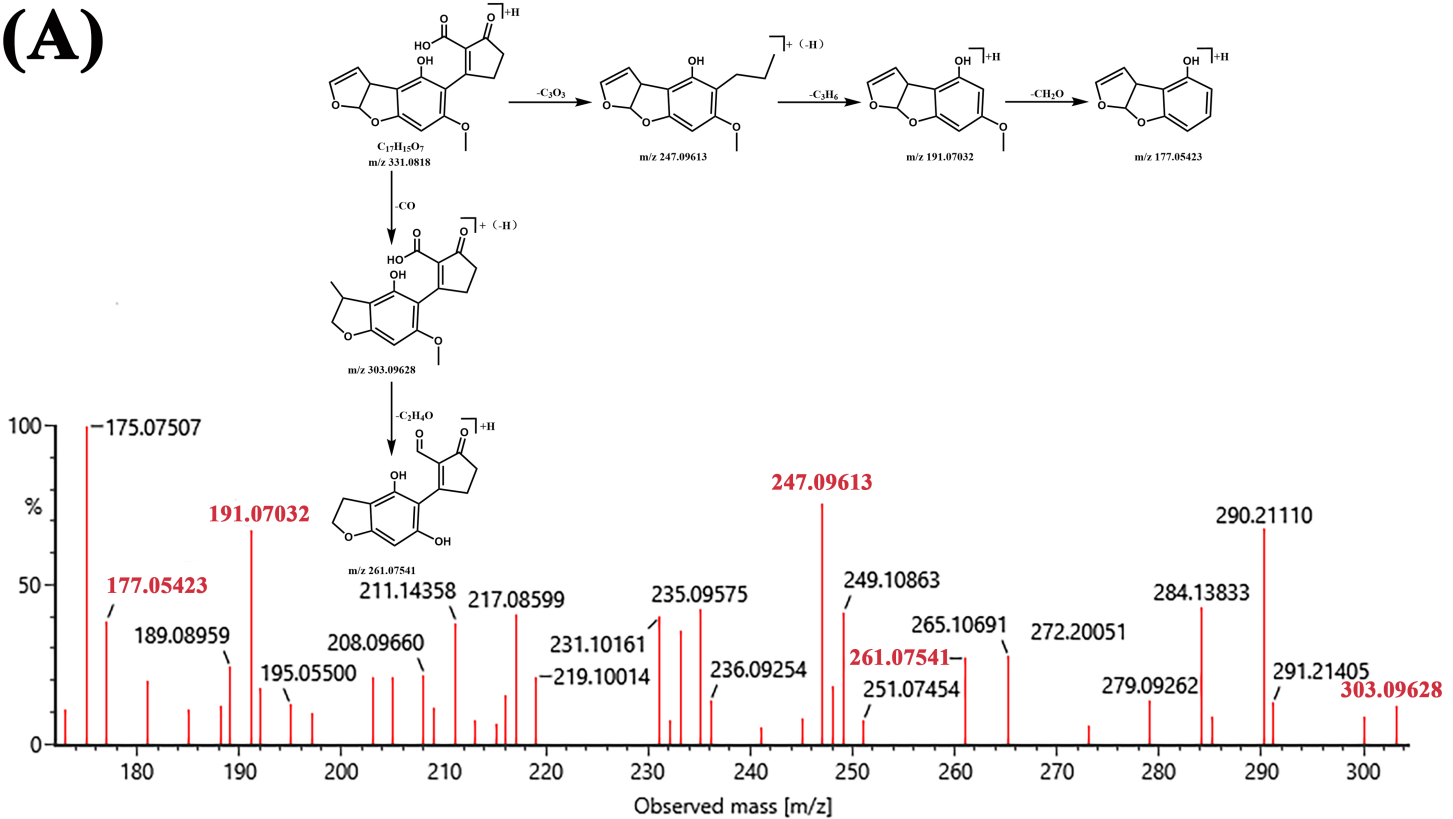

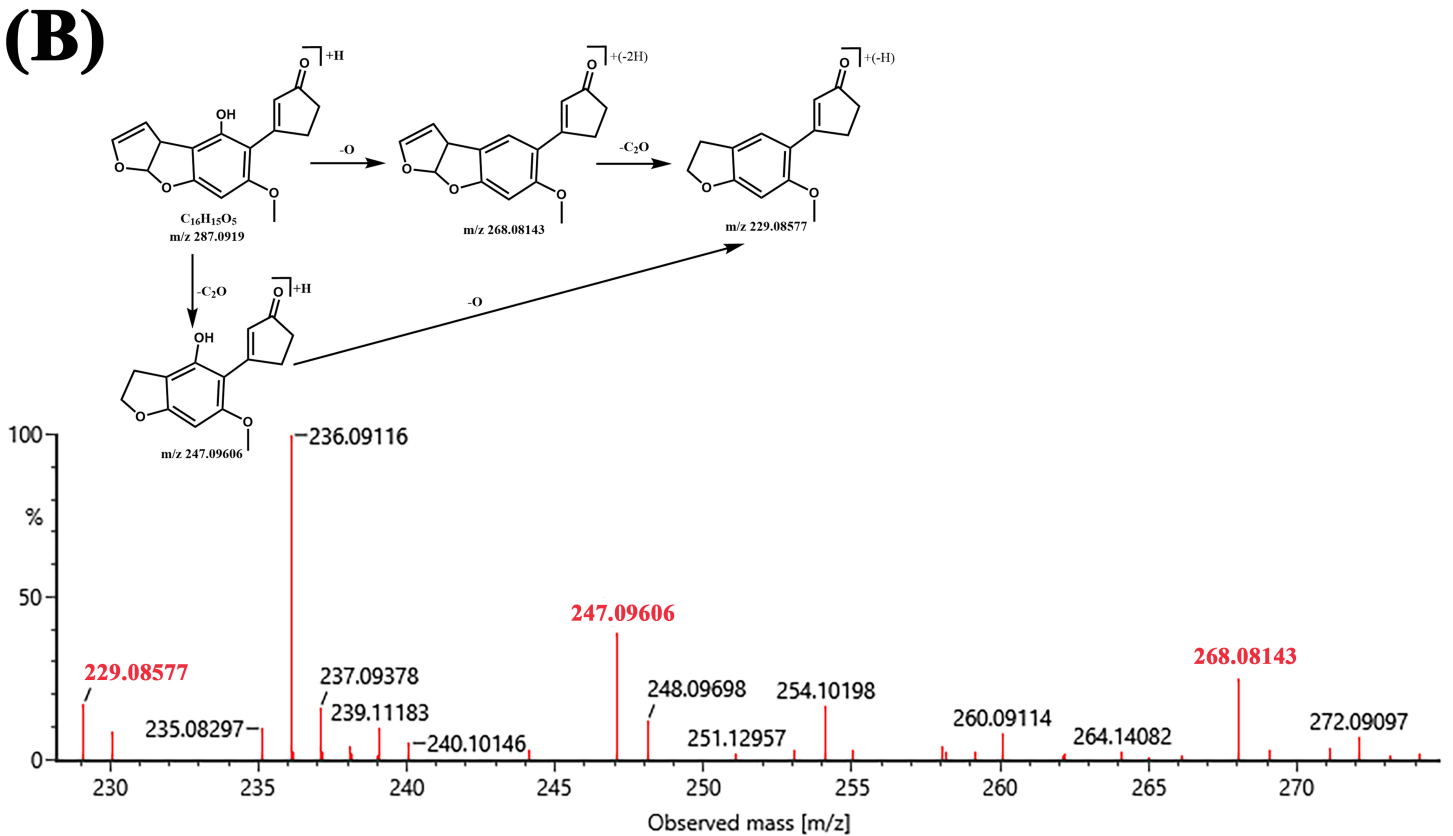

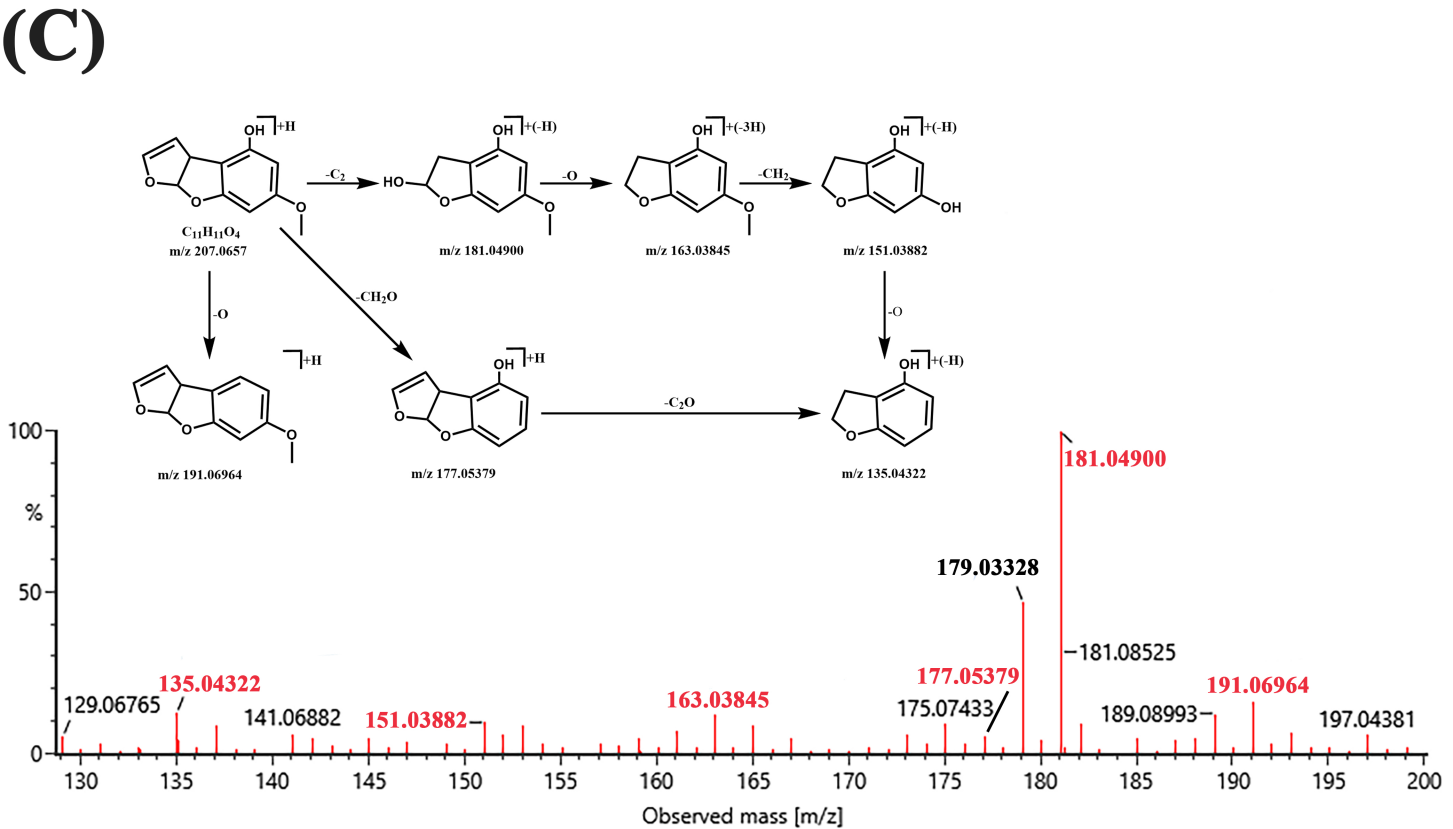

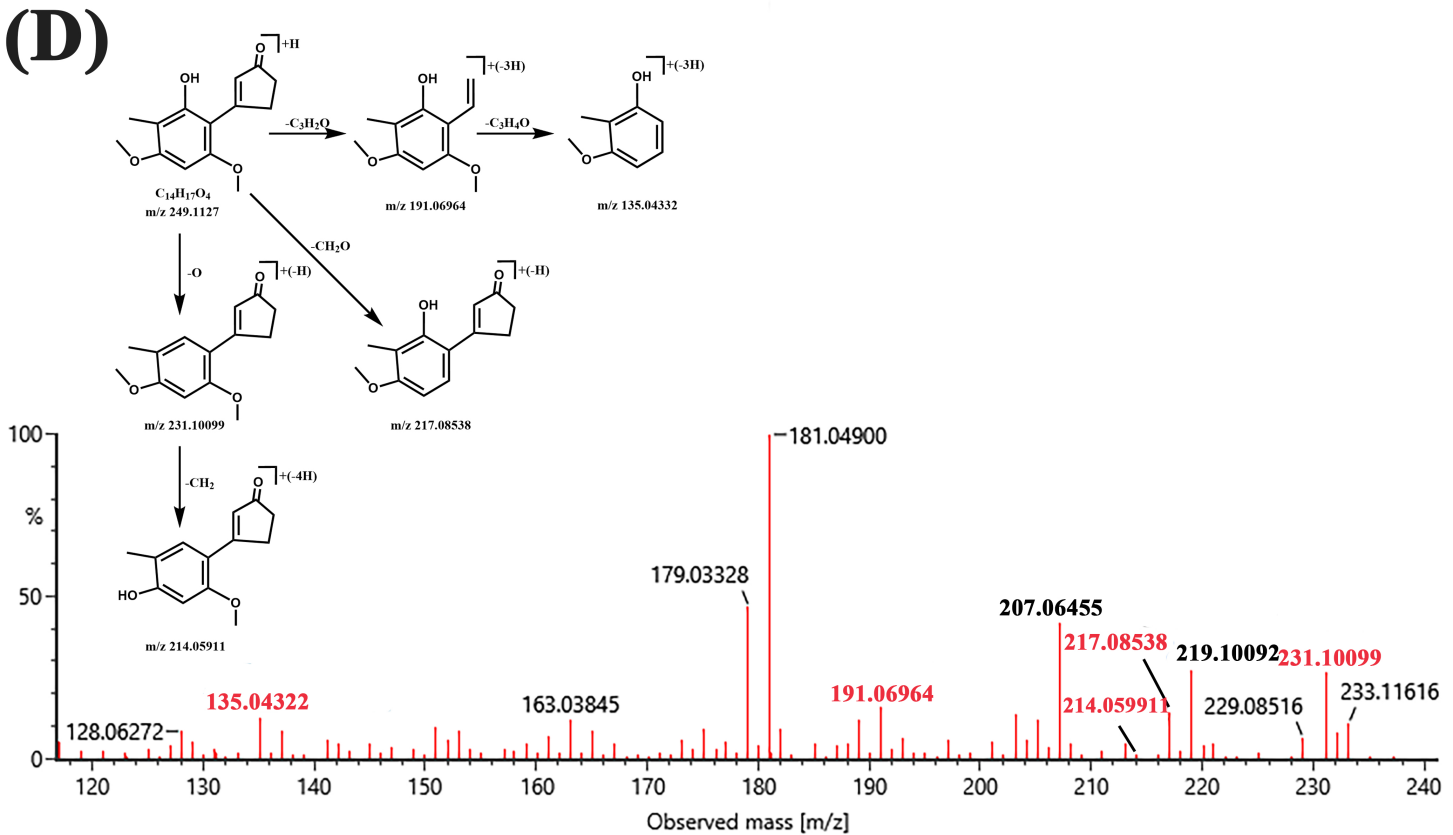

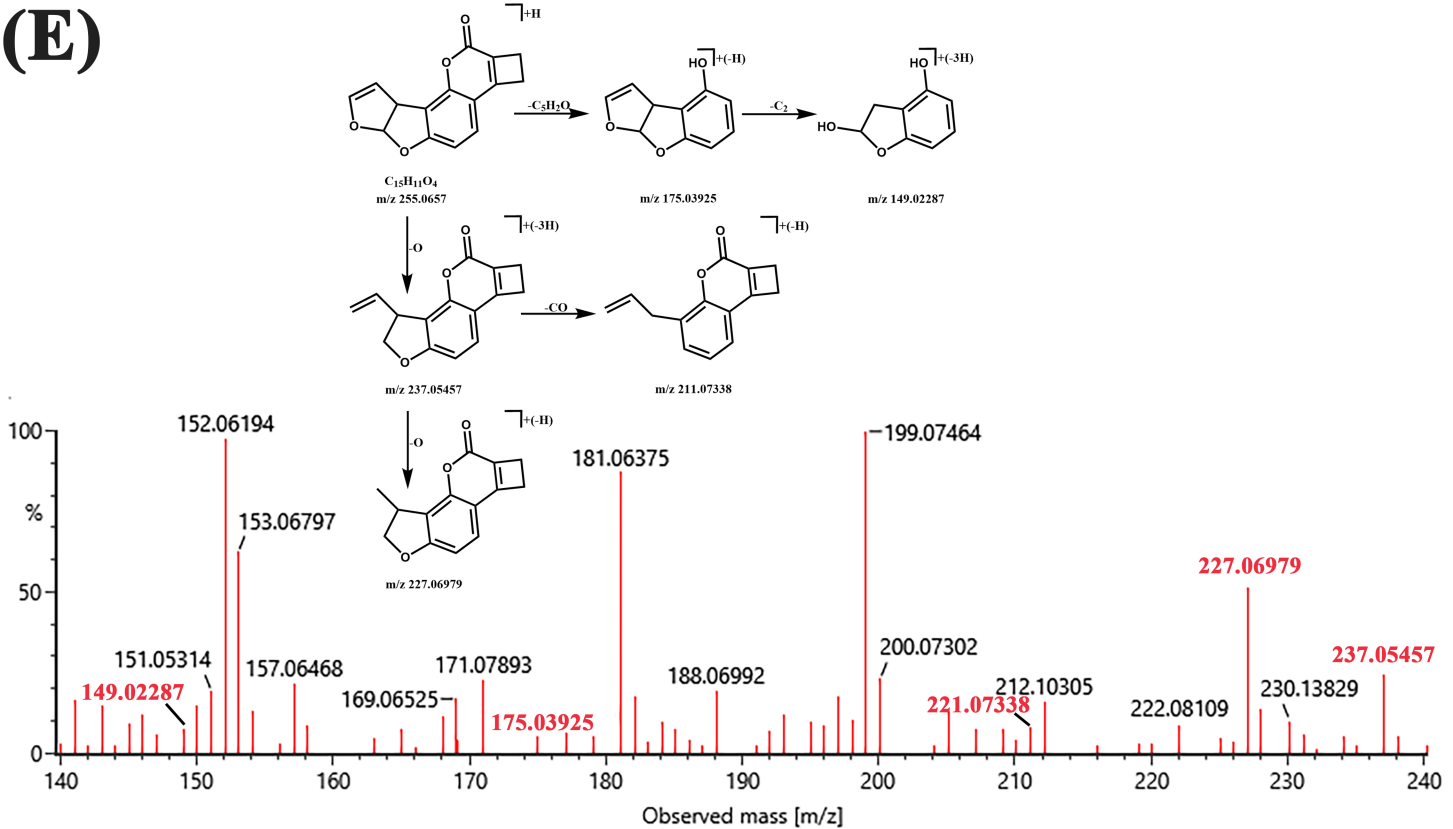

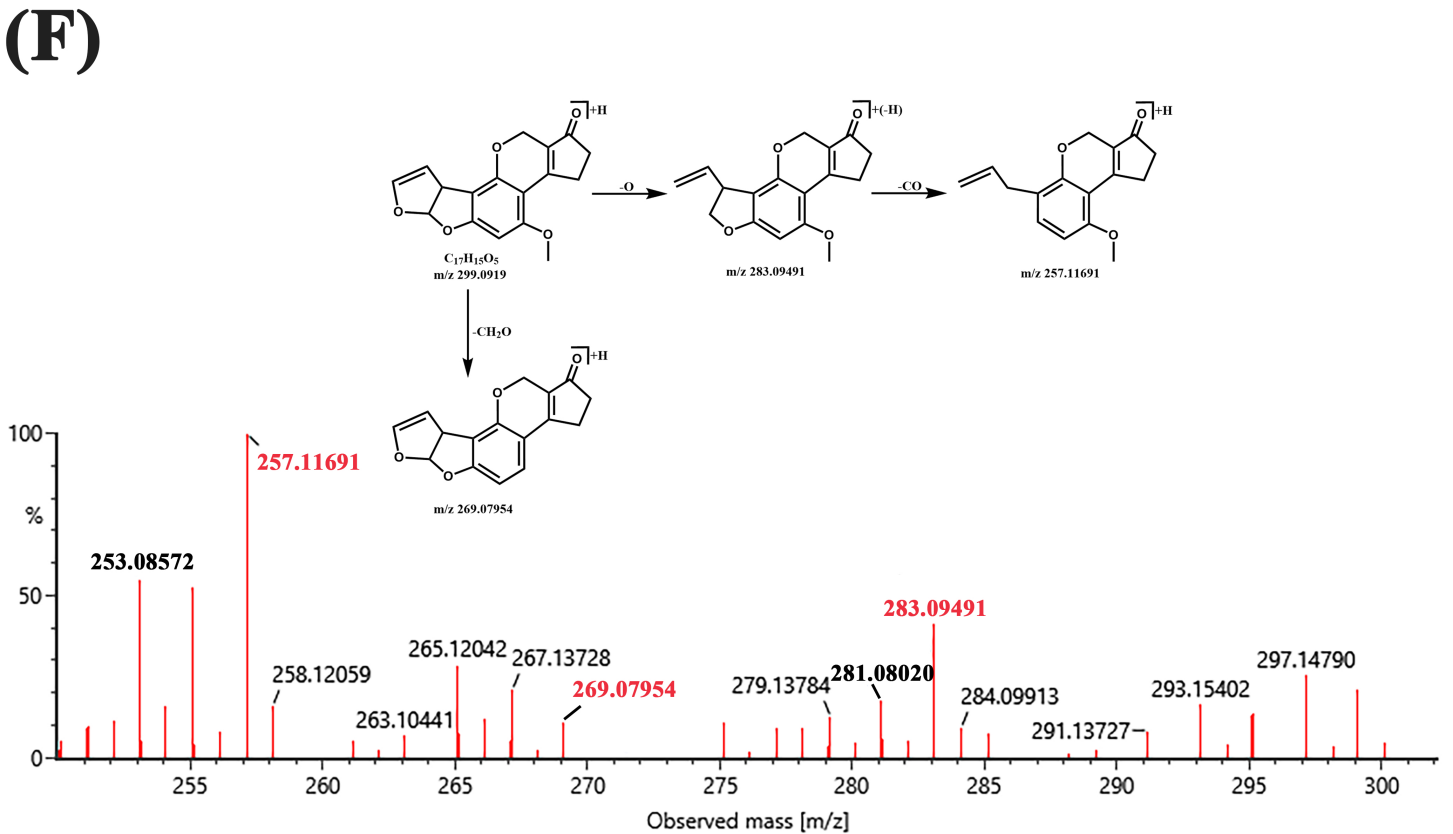
UPLC Q-TOF MS (electrospray ionization source positive ionization mode) spectrum of AFB_1_ and its six key degradation products with insets showing their fragmentation pathways. Panels (**A**–**F**) correspond to the six key degradation products: (**A**) C_17_H_15_O_7_; (**B**) C_16_H_15_O_5_; (**C**) C_11_H_11_O_4_; (**D**) C_14_H_17_O_4_; (**E**) C_15_H_11_O_4_; (**F**) C_17_H_15_O_5_.

Using the structural analysis of AFB_1_ as an example (Figure 4), under low collision energy (5 eV) in positive ion (PI) mode, AFB_1_ produced precursor ions ([M + H]^+^, *m*/*z* 313.0712); under high collision energy (40 eV) conditions, AFB_1_ yielded a series of fragment ions with continuous characteristics, mainly including *m*/*z* 299.09052, 285.07556, 271.09787, 247.09618, 205.04851, 191.07001, 179.03328, and 163.03748. The possible structural formulas were analyzed based on relevant information of AFB_1_ in Table 4 and imported in MOL format into MassFragment, which performs structural analysis and characteristic value assignment for fragment ions. By integrating fragment ion data, fragment ion matching results actually measured by Q-TOF MS, and theoretical mass numbers with experimentally determined mass data, the most probable structures of the degradation products were ultimately determined. Figure 4 shows the fragmentation pathway diagram of AFB_1_ and its key degradation products obtained by this method.

**Figure 5:**
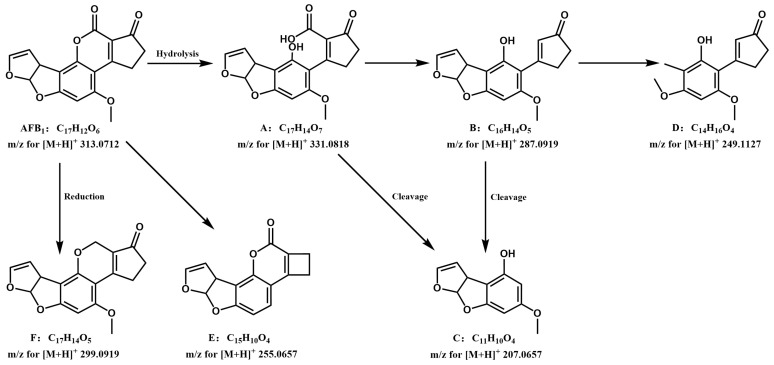
Hypothetical degradation pathways of AFB_1_ by *T. reesei* GG-T40.

Figure 5 shows the structures and degradation pathways of all degradation products of AFB_1_. Its lactone ring is first hydrolyzed to generate the key carboxylic acid intermediate AFB_1_-a (A: C_17_H_14_O_7_). This intermediate can be transformed via two main pathways: on one hand, AFB_1_-a can undergo direct decarboxylation to produce AFD_1_ (B: C_16_H_14_O_5_). On the other hand, it can also undergo direct hydrolysis, leading to cleavage of the cyclic peptide ketone ring and the formation of AFD_2_ (C: C_11_H_10_O_4_). These transformations are consistent with the enzymatic mechanism elucidated by Taylor et al. [46]: the F420H2-dependent reductase families (FDR-A and FDR-B) they discovered in Mycobacterium smegmatis can utilize the coenzyme F420H2 to reduce the α,β-unsaturated ester bond in the AFB_1_ molecule; the resulting reduction product is chemically unstable and subsequently undergoes spontaneous hydrolysis and decarboxylation, thereby driving the aforementioned conversion of AFB_1_-a to AFD_1_ or AFD_2_. Subsequently, AFD_1_ generated via the decarboxylation pathway can serve as a key intermediate for further reactions: it can undergo hydrolysis leading to cleavage of the bond connected to the cyclic peptide ketone ring, also generating AFD_2_ [47]; or it can be converted to C_14_H_16_O_4_ (D) via elimination of the C8–C9 furan double bond. Additionally, the cyclopentenone ring underwent ring-opening to form C_15_H_10_O_4_ (E), while AFB_1_ itself could also be reduced to C_17_H_14_O_5_ (F).

### 3.4. Toxicity Analyses of Degradation Products of AFB_1_

The toxic sites of AFB_1_ include the C8–C9 furan double bond, the coumarin lactone ring structure, and the cyclopentenone ring [48]. Its toxic mechanism involves CYP450-mediated specific epoxidation of the C8–C9 double bond under the influence of the cyclopentenone ring, generating AFBO, with the highly reactive exo-AFBO being the primary toxic form [49]. Leveraging the planarity provided by the coumarin ring, the activated molecule can intercalate between DNA base pairs. Simultaneously, exo-AFBO initiates a nucleophilic attack at the N7 position of guanine via an SN2 mechanism, forming a covalent adduct (AFB_1_-N7-Gua) [50]. This covalent adduct may drive point mutations that potentially initiate carcinogenesis [51,52], induce DNA strand breaks, or undergo hydrolysis to form AFB_1_-dihydrodiol, which binds to various proteins, causing tissue damage, inflammation, and hyperproliferation, ultimately leading to carcinogenic responses. Among the degradation products generated by the action of *T. reesei* GG-T40 on AFB_1_, the lactone ring of AFB_1_ was disrupted in products A, B, C, D, and F, and the cyclopentenone ring was disrupted in product E. Notably, C_14_H_16_O_4_ (D) simultaneously lost two critical toxicophores (the furan double bond and the coumarin lactone ring). Consequently, the toxicity of these six degradation products was significantly reduced or eliminated.

In our previous study [21], analysis of the potential detoxification components of *T. reesei* CGMCC3.5218 revealed that its culture supernatant achieved a degradation rate of 91.8% for AFB_1_. Further research indicated that a heat-stable enzyme or protein likely played a major role in the degradation process. These findings were consistent with the results of Xu Yanhua et al. [53], who identified and confirmed that *P. aeruginosa* M-4 disrupts three toxic sites of AFB_1_, including the lactone ring, by producing a heat-stable extracellular enzyme. The study by Haocheng Liu et al. [54] also discovered that *Rhodococcus turbidus* PD630 produces an extracellular enzyme that efficiently degrades AFB_1_ by cleaving its lactone ring and converting it into entirely distinct compounds, and explored the optimal conditions for this enzyme’s activity. Furthermore, Wang, X. et al. [55] extracted and purified a high-purity enzyme from *Myroides odoratimimus* (3J2MO), which achieved a 95% degradation rate for AFB_1_, while elucidating its mechanism involving lactone ring cleavage and epoxide hydrolysis. Based on these studies and further analysis of the degradation products, we speculate that *T. reesei* GG-T40 produces an enzyme or enzymes that specifically recognize and cleave the lactone ring bond. This broad substrate adaptability suggests that the strain or its enzyme preparations hold significant application potential in simultaneously degrading various mycotoxins whose toxicity core is the lactone ring.

### 3.5. Structural Formulas of ZEN Degradation Products

**Figure 6:**
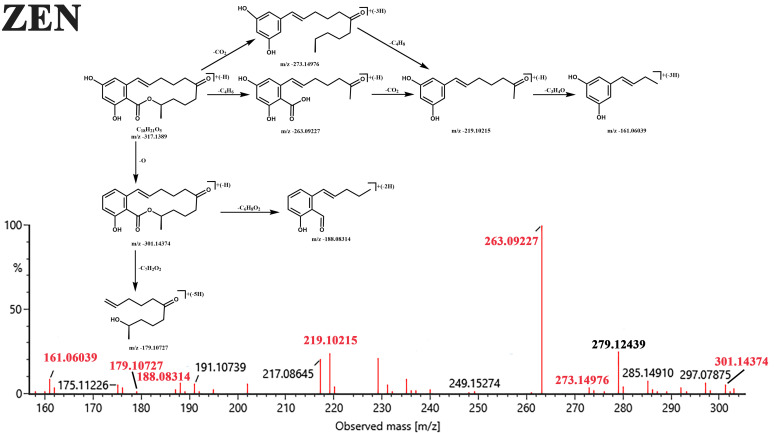

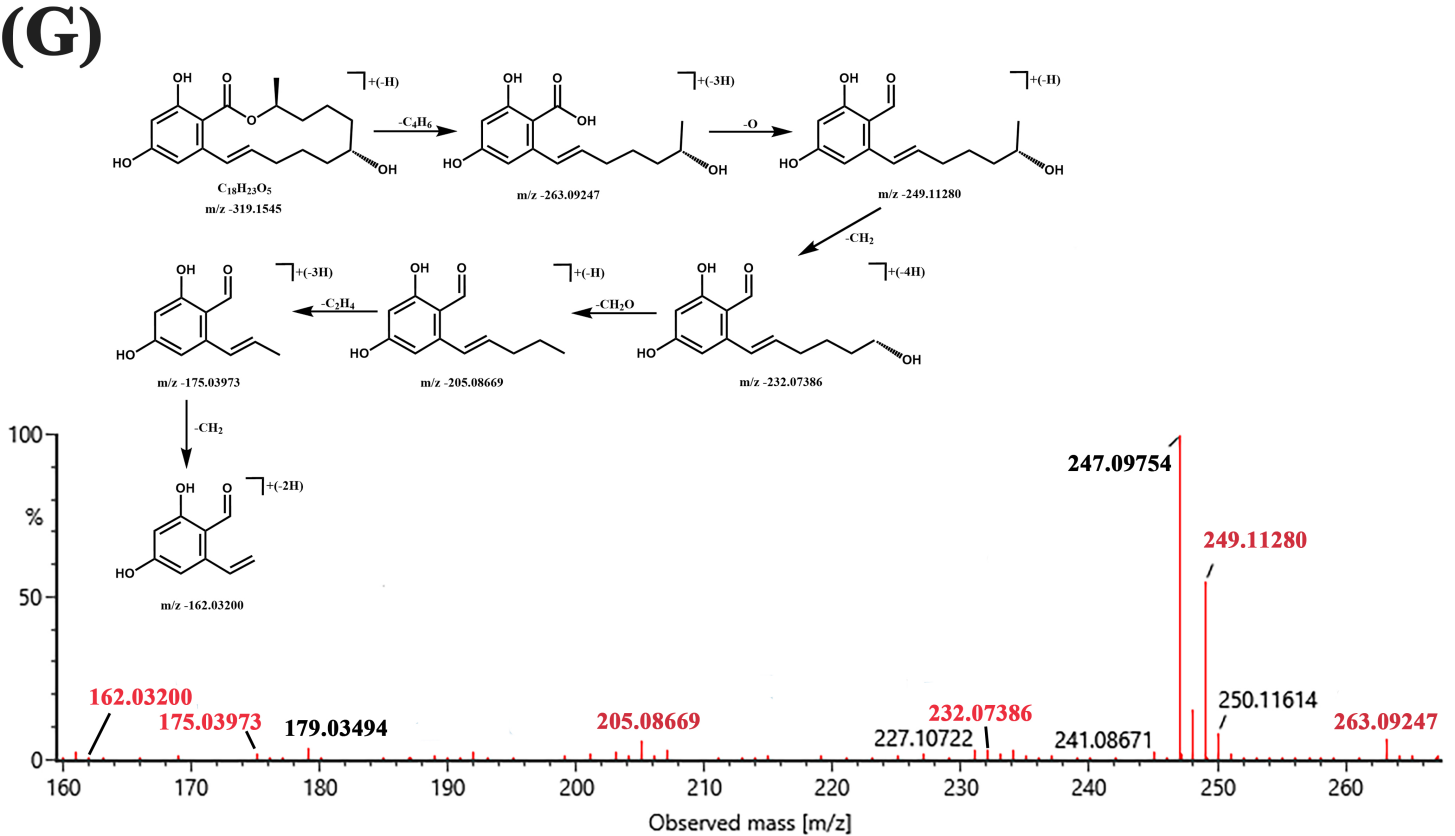
UPLC Q-TOF MS (electrospray ionization source negative ionization mode) spectrum of ZEN and its key degradation products G (C_18_H_24_O_5_) with insets showing their fragmentation pathways.

The transformation of ZEN primarily involves hydrolysis, reduction, and functional group modifications of its Lactone ring, C-4 hydroxyl group [56]. MassLynx V4.1 calculated the molecular formula of the ZEN degradation product as C_18_H_24_O_5_ (319.1545 *m*/*z* [M − H]^−^) based on Q-TOF MS data. The decrease of DBE compared to ZEN indicates an addition reaction occurred during degradation. Via comparison of chromatographic retention times and multistage mass spectral fragment ions with reference standards of this degradation product, combined with structural interpretation by MassFragment software based on accurate mass data, the degradation product was identified as α-/β-zearalenol. Figure 6 illustrates the fragmentation pathways of ZEN and its degradation product G (α-/β-ZOL).

**Figure 7:**
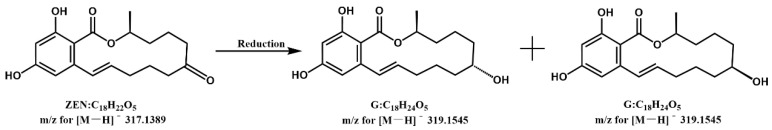
Hypothetical degradation pathways of ZEN by GG-T40.

As shown in Figure 7, ZEN features a C6′-keto carbonyl group that is susceptible to reduction, leading to the formation of reduced products with the molecular formula C_18_H_24_O_5_, namely α- or β-zearalenol (α-/β-ZOL).

### 3.6. Toxicity Analyses of Degradation Products of ZEN

The toxicity of ZEN primarily originates from the structural motif comprising the lactone ring and the C4-hydroxy group, which closely resembles endogenous estrogen 17β-estradiol in both spatial configuration and polarity distribution. This structural similarity enables ZEN to competitively bind to estrogen receptors, mimicking estrogenic physiological effects [57], thereby disrupting endocrine homeostasis and contributing to reproductive dysfunction and related disorders [58].

In this study, the degradation of ZEN did not involve cleavage or destruction of the lactone ring, but rather proceeded via stereoselective reduction at the C6′position to yield α-/β-ZOL. Consequently, the degradation products retain certain toxicity. It has been well documented that α-ZOL and β-ZOL are major reduced metabolites of ZEN in both in vivo and in vitro settings [59]. Notably, although these two compounds are isomers, they exhibit significant differences in estrogenic activity and toxicity: α-ZOL demonstrates stronger estrogenic effects than ZEN, whereas β-ZOL is significantly less active [60]. This divergence stems primarily from the distinct stereochemical configuration of the C6′-hydroxyl group: in α-ZOL, the C6′-OH and C7′-H adopt a trans conformation, which confers an overall molecular shape and hydrogen-bonding pattern more similar to 17β-estradiol, thereby enabling higher affinity and binding stability to estrogen receptors.

Therefore, when evaluating the detoxification efficacy of *T. reesei* GG-T40 against ZEN, merely measuring the residual level of ZEN is insufficient for a comprehensive safety assessment. It is essential to separately quantify α-ZOL and β-ZOL. Optimizing chromatographic separation conditions or employing technical approaches such as nuclear magnetic resonance (NMR) spectroscopy to achieve accurate identification and quantification of both isomers will contribute to a more precise assessment of the combined toxic effects and provide a more scientific basis for food safety and toxicological research.

**Figure 8:**
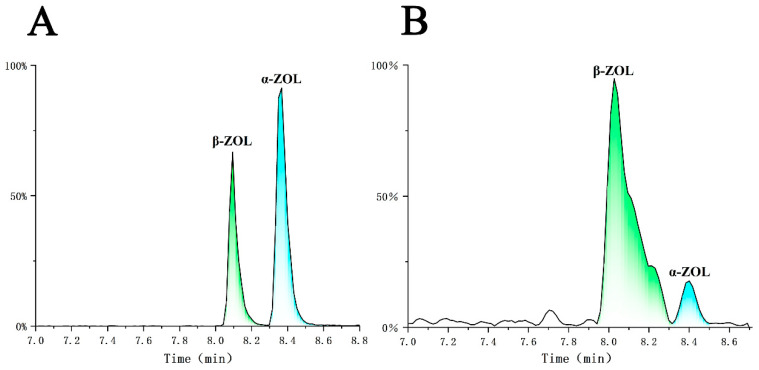
Chromatographic separation and quantitative analysis of ZEN degradation products—α-ZOL and β-ZOL. (**A**) HPLC chromatogram of standards of α-ZOL and β-ZOL. (**B**) HPLC chromatogram of the experimental sample showing the degradation products.

Therefore, it is essential to further quantitatively analyze its transformation products, α-ZOL and β-ZOL, when evaluating the detoxification effect of *T. reesei* GG-T40 on ZEN. By further optimizing the chromatographic separation conditions, we achieved accurate identification and quantification of these two isomers (Figure 8). The results indicated that the concentration of α-ZOL in the experimental group was 4.5 ng/mL, while the concentration of β-ZOL was 48.9 ng/mL. We speculate that during the co-culture stage, α-ZOL and β-ZOL may undergo conjugation reactions with endogenous sulfonic or glucuronic acids produced by *Trichoderma* metabolism. This process would significantly alter their molecular weight, polarity, and mass spectrometry fragmentation behavior, leading to the generation of conjugated metabolites that fall outside the detection scope of our method. This could be the main reason why the detected concentrations of the free-form products were significantly lower than the theoretical values. Anyhow, the proportion of β-ZOL among the degradation products was 91.5%, which was significantly higher than that of α-ZOL. Meanwhile, it has been reported in the literature that α-ZOL and β-ZOL can be further reduced to α-ZAL and β-ZAL, and the latter can be interconverted or metabolized to a lesser extent into ZAN [61]. However, during the one-year monitoring period in this study, no such subsequent transformation events were observed. Specifically, no α-ZAL, β-ZAL, or ZAN was detected, and the concentrations of the residual ZEN and its initial reduction products (α-ZOL and β-ZOL) remained stable. This result indicates that under the experimental conditions, the degradation products of ZEN did not undergo further transformation into other known toxic derivatives. The species-specific conversion rate of ZEN to α-ZOL can be regarded as a bioactivation reaction, while conversion to β-ZOL may be considered a detoxification reaction [62]. Thus, it can be concluded that the degradation of ZEN by *T. reesei* GG-T40 significantly reduces its toxicity. Nevertheless, this study provides important evidence for a more scientific assessment of the toxicological effects of ZEN detoxification by *T. reesei* GG-T40. Critically, detoxification should be confirmed through two main approaches: (1) Detecting and identifying degradation products, followed by structural analysis and comparison with existing literature to assess whether these products are less toxic than the parent compound. (2) Performing one or more toxicity assays using specific organisms or cell lines to verify reduced toxicity after degradation. Undoubtedly, a combination of both methods provides the most comprehensive assessment of detoxification. Therefore, it remains essential to further validate the toxicity of the products after ZEN degradation by strain *T. reesei* GG-T40 using specific organisms or cell lines.

### 3.7. In Vivo Toxicity Assessment of Degradation Products

To evaluate the in vivo safety of the degradation products of AFB_1_ and ZEN by *T. reesei* GG-T40, this study conducted acute oral toxicity tests and intraperitoneal injection pathogenicity tests in mice. The results (Table 6) showed that the degradation products did not exhibit any significant toxicity or pathogenicity in the aforementioned tests. In the acute oral toxicity test, after a single oral gavage of degradation products at a dose as high as 5000 mg/kg, no deaths occurred during the 14-day observation period, and normal weight gain was observed in both female and male mice. According to the acute toxicity dose classification standard in the “General Biosafety Standard for Microbial Fertilizers (NY/T 1109-2017) [63]”, the degradation products of AFB_1_ and ZEN after treatment belong to the “practically non-toxic” category. In the intraperitoneal injection pathogenicity test, all test mice survived during the 14-day experimental period. Subsequent gross and histopathological examinations conducted on days 3, 7, and 14 showed no significant organ lesions, demonstrating that the degradation products are non-pathogenic and non-irritating. Therefore, the in vivo toxicity assessment results fully demonstrate that the degradation products of AFB_1_ and ZEN by *T. reesei* GG-T40 possess high biosafety, which is consistent with our toxicity predictions based on the structure of the degradation products. *T. reesei* GG-T40 achieves efficient detoxification by converting highly toxic AFB_1_ and ZEN into low-toxicity or non-toxic products, providing key scientific evidence for its use as a safe and reliable biological detoxifying agent in the control of mycotoxin contamination in grains and feed.

## 4. Conclusions

This study elucidates the degradation effects and molecular mechanisms of the newly screened *Trichoderma reesei* GG-T40 strain on aflatoxin B_1_ (AFB_1_) and zearalenone (ZEN). The degradation rates of AFB_1_ and ZEN by this strain reached 98.6% and 88.4%, respectively. Based on UPLC Q-TOF-MS analysis, six AFB_1_ degradation products and two ZEN degradation products were deduced, and the toxicity changes of each product were further evaluated based on structure–activity relationships. The results showed that the coumarin lactone ring and the cyclopentenone ring of AFB_1_ were cleaved. The disruption of these key toxic structures led to significantly reduced or completely eliminated toxicity. ZEN was mainly converted into α-ZOL and β-ZOL derivatives. Although its lactone ring remained unopened, retaining partial estrogenic activity, 91.5% of the products were β-ZOL with lower estrogenic activity, while only 8.5% were α-ZOL with higher activity, resulting in an overall effective reduction in toxicity. Furthermore, in vivo toxicological experiments demonstrated that the degradation products were non-toxic and non-pathogenic, and this result was consistent with the structural prediction conclusions, jointly verifying the safety of the detoxification process. This study is the first to comprehensively reveal the transformation pathways and product profiles of AFB_1_ and ZEN degradation by *T. reesei* GG-T40, and systematically evaluate the toxicological safety of its degradation products based on in vivo and in vitro evidence. It provides a solid data foundation and theoretical support for the application of this strain as an environmentally friendly biological detoxifying agent in the control of mycotoxin contamination in grains and feed raw materials, and holds significant importance for controlling mycotoxin contamination, ensuring the quality and safety of grain products, and promoting the development of green agriculture.

## 5. Application Prospects and Challenges

Although this study demonstrates that *Trichoderma reesei* GG-T40 possesses efficient and safe degradation capabilities against AFB_1_ and ZEN under controlled laboratory conditions, several challenges must be overcome for its practical application. Firstly, in complex matrices such as whole grains and mixed feed, components like proteins, starches, and fats may inhibit the contact efficiency and activity of the fungal cells or enzymes, while physical barriers (e.g., seed coats) could also impede degradation efficacy. Therefore, future research urgently needs to validate the degradation performance of *T. reesei* GG-T40 in actual contaminated agricultural products like maize and wheat. Secondly, industrial application faces challenges including scaled fermentation production, formulation stability, application cost, and regulatory compliance.

To address these challenges and promote the practical application of this technology, the following directions for future research are proposed:

1. Elucidating the Molecular Basis and Regulatory Networks of the Degradation Mechanism: Applying multi-omics technologies to analyze the expression and regulatory networks of key genes involved in *T. reesei* GG-T40’s degradation of AFB_1_ and ZEN, clarifying the molecular basis of its efficient and simultaneous degradation, and providing a foundation for genetic improvement of the strain.
2. Preparation and Application of Key Degradative Enzyme Systems: Isolating and identifying the key enzymes responsible for degrading AFB_1_ and ZEN in *T. reesei* GG-T40, and preparing high-purity enzymes or multi-enzyme complexes via heterologous expression to achieve precise, efficient, and stable detoxification.
3. Performance Optimization in Complex Matrices: In simulated or genuinely contaminated feed/food matrices, optimizing the application method of the fungal agent and exploring combined strategies with specific probiotics or physical methods to enhance its degradation efficiency and stability in complex environments.
4. Development of Scalable Production Processes: Leveraging the well-established industrial fermentation background of *T. reesei* to develop processes suitable for the large-scale production of highly active fungal agents or enzyme formulations, and establishing corresponding quality control standards.

## Figures and Tables

**Table 1:** Mycobacterium species and sources.

Species ^(a)^	Strain ^(b)^	Geographical Origin	Source
*T. asperellum*	GC-T2	Tibet, China	Soil
	GC-T4	Tibet, China	Soil
*T. atroviride*	GC-T78	Yunnan, China	Rotten wood in soil
	GC-T8	Tibet, China	Soil
	GC-T9	Tibet, China	Soil
*T. citrinoviride*	GC-T19	Tibet, China	Soil
	GC-T20	Tibet, China	Soil
	GC-T21	Tibet, China	Soil
*T. dorotheae*	GC-T24	Tibet, China	Soil
	GC-T25	Tibet, China	Soil
*T. erinaceum*	GC-T27	Tibet, China	Soil
*T. gamsii*	GC-T26	Tibet, China	Soil
*T. harzianum*	GC-T40-1	Tibet, China	Soil
	GC-T41	Tibet, China	Soil
*T. hispanicum*	GC-T42	Tibet, China	Soil
*T. inhamatum*	GC-T44	Tibet, China	Soil
*T. koningii*	GC-T75-1	Yunnan, China	Rotten wood in soil
*T. longifialidicum*	GC-T87	Tibet, China	Soil
*T. reesei*	GG-T40	China	Rotten wood in soil
*T. sulphureum*	GC-T43-1	China	Rotten wood in soil
*T. velutinum*	GC-T82	Tibet, China	Soil
	GC-T83	Tibet, China	Soil
	GC-T85	Tibet, China	Soil
*T. viride*	GC-T45-1	Shandong, China	Soil
*T. virilente*	GC-T88	Tibet, China	Soil
	GC-T89	Tibet, China	Soil

^(a)^ Species identified by sequence analysis of ITS-1 and ITS-2. ^(b)^ Strains were named by our laboratory.

**Table 2:** Mobile phase gradient.

Time (min)	Flow (mL/min)	A (%)	B (%)
0.00	0.400	95	5
0.50	0.400	95	5
5.50	0.400	50	50
9.00	0.400	5	95
10.50	0.400	5	95
12.00	0.400	95	5

**Table 3:** Mobile phase gradient.

Time (min)	Flow (mL/min)	A (%)	B (%)
0.01	0.400	90	10
2.00	0.400	90	10
2.50	0.400	70	30
3.50	0.400	30	70
5.00	0.400	10	90
9.00	0.400	10	90
9.50	0.400	90	10

The system is programmed to stop at 11.00 min.

**Table 4:** Mass accuracy measurement using UPLC Q-TOF MS for the AFB_1_ and degradation products of AFB_1_.

Compound	Retention Time (min)	Formula	Calculated Mass (*m*/*z*) ^a^	Experimental Mass (*m*/*z*) ^a^	Mass Error		DBE
					mDa	PPM	
AFB_1_	4.665	C_17_H_13_O_6_	313.0712	313.0754	4.2	13.4	11.5
A	3.288	C_17_H_15_O_7_	331.0818	331.0800	−1.8	−5.4	10.5
B	4.330	C_16_H_15_O_5_	287.0919	287.0937	1.8	6.3	9.5
C	7.679	C_11_H_11_O_4_	207.0657	207.0706	4.9	23.7	6.5
D	7.665	C_14_H_17_O_4_	249.1127	249.1151	2.4	9.6	6.5
E	3.994	C_15_H_11_O_4_	255.0657	255.0665	0.8	3.1	10.5
F	6.585	C_17_H_15_O_5_	299.0919	299.0936	1.7	5.7	10.5

^a^ All the *m*/*z* in our experiment is the *m*/*z* of [M + H]^+^.

**Table 5:** Mass accuracy measurement using UPLC Q-TOF MS for the ZEN and degradation products of ZEN.

Compound	Retention Time (min)	Formula	Calculated Mass (*m*/*z*) ^b^	Experimental Mass (*m*/*z*) ^b^	Mass Error		DBE
					mDa	PPM	
ZEN	6.298	C_18_H_21_O_5_	317.1389	317.1396	0.7	2.2	8.5
G	6.987	C_18_H_23_O_5_	319.1545	319.1563	1.8	5.6	7.5

^b^ All the *m*/*z* in our experiment is the *m*/*z* of [M − H]^−^.

**Table 6:** Acute oral toxicity test and intraperitoneal pathogenicity test of AFB_1_ and ZEN degradation products by *T. reesei* GG-T40. (N/A: Not applicable).

Toxicity Test Type	Dose	Animal No.	Initial Weight (g)	Final Weight (g)	Mortality	Gross Pathology (Day 3/7/14)	MTD/LD_50_
Acute Oral Toxicity	5000 mg/kg	20	15.8 ± 0.5	31.1 ± 0.8	0/20	N/A	>5000 mg/kg
	5000 mg/kg	20	17.0 ± 0.3	33.5 ± 0.4	0/20	N/A	>5000 mg/kg
Pathogenicity	0.50 mL	15	20.4 ± 0.6	N/A	0/15	No lesions	N/A
	0.50 mL	15	22.7 ± 0.7	N/A	0/15	No lesions	N/A

## Data Availability

The original contributions presented in this study are included in the article. Further inquiries can be directed to the corresponding authors.
